# Recent Advancements in Carbon Nano-Infused Cementitious Composites

**DOI:** 10.3390/ma14185176

**Published:** 2021-09-09

**Authors:** Eryk Goldmann, Marcin Górski, Barbara Klemczak

**Affiliations:** Department of Structural Engineering, Faculty of Civil Engineering, Silesian University of Technology, 44-100 Gliwice, Poland; marcin.gorski@polsl.pl (M.G.); barbara.klemczak@polsl.pl (B.K.)

**Keywords:** cementitious composites, cement nanocomposites, carbon nanomaterials, carbon nanotubes, graphene, smart materials, structural health monitoring

## Abstract

A rising demand for efficient functional materials brings forth research challenges regarding improvements in existing materials. Carbon infused cementitious composites, regardless of being an important research topic worldwide, still present many questions concerning their functionality and properties. The paper aims to highlight the most important materials used for cementitious composites, their properties, and their uses while also including the most relevant of the latest research in that area.

## 1. Introduction

The term ‘nanotechnology’ was first defined by professor Norio Toguchi in 1974. Defining the process of modifying material on an atomic level. It had continued an idea put forward by American physicist Richard Feynman, who said in 1959: “There’s plenty of room at the bottom”. With time, a basic definition of nanotechnology has evolved to be finally determined as: “the application of scientific knowledge to manipulate, control, and restructure matter at the atomic and molecular level in the range of 1–100 nm to exploit size-dependent and structure-dependent properties and phenomena distinct from those at different scales” [1].

Main opportunities for the use of nanotechnology were at first seen in the fields of electrical engineering and biotechnology. Since the beginning of the XXI century, nanotechnology also attracts researchers associated with civil engineering, and the founding of the research in this area is still rising [2]. The main scope of research in the case of cementitious nanocomposites is creating materials with enhanced mechanical properties as well as added functionality. Resistance to ageing and environmental aggression; capability of auto-monitoring or auto-repair; and energy saving through energy harvesting are important factors to consider regarding more resilient structures and concepts of smart cities. Moreover, resilient materials can effectively reduce the amount of maintenance needed while sensors inbuilt in building structures will allow for safer exploitation and early detection of problems. These materials are often called ‘smart materials’, as they possess properties that are not observed in a usual form of such material. Most often, the smartness of the material is connected with abovementioned functionality. For example, the ability to self-sense damage and strains is compared to a living organism’s neural system. Such form of biomimetic behavior is one of the most popular and most researched properties for a variety of smart materials.

Materials based on Portland cement are still the most popular type of materials used in civil engineering structures. Possessing high compressive and dynamic resistance, they are still prone to cracking and are generally brittle materials. Cracks caused by either mechanical or non-mechanical factors might often lead to degradation of materials, reduce their tightness, and allow for infiltration of aggressive substances. Fiber-reinforced concretes seem to counteract the problem of cracking; however, they are not able to bridge cracks at the nanoscale where they originate from. The addition of nanomaterials to concrete allows for strengthening at the molecular scale, bridging those small-scale cracks and densifying the material’s nanostructure. Moreover, nanoparticles allow for accelerated growth of stronger C-S-H phase crystals, increasing the material’s strength also in the matrix.

First known applications of nanoparticles in concrete used mostly titanium, silica, and aluminium oxides. Those additions have been proven to increase concrete’s extreme temperature resistance and intensify the hydration process [3,4,5,6,7]. Further usage of various types of carbon nanomaterials came mainly from the desire to add functionality to the composites. The idea of smart material sensors and heat transferring materials emerged, with the former being already used in aircraft engineering and epoxy composites.

With regard to their form and shape, nanomaterials can be classified as follows [8]:

- Zero-dimensional materials (0D)—singular nanoparticles that mainly influence properties of the cementitious matrix itself,
- One-dimensional materials (1D)—elongated structures (nanotubes, nanofibers), with one dimension much bigger than the other two that can bridge cracks,
- Two-dimensional materials (2D)—platelets, for example, graphene, which two dimensions are much bigger than the third one, with a large surface area and ability to bridge cracks.

The presented paper focuses on carbon-based nanomaterials, their properties, and applications mainly in smart materials. The paper is organized as follows: Section 2 describes the most commonly used types of carbon-based nanomaterials and their general properties; Section 3 presents how those properties affect cementitious composites; Section 4 discusses the problem of dispersion of nanomaterials; Section 5 shows examples of the practical application of nano-carbon cementitious composites; Section 6 discusses known attempts on creating computational models for nanocomposites; Section 7 concludes the paper.

## 2. Carbon Nanomaterials

Carbon-based nanomaterials are atomic structures built from amorphous types of carbon, mainly fullerenes and graphite. They are composed of crystalline structures with strong bonds between carbon atoms. Depending on the arrangement and combination of those structures, they can form one- or two-dimensional formations. In general, carbon nanomaterials can come in each of the dimensional forms mentioned in the previous section.

### 2.1. Graphene

Graphene, a two-dimensional material, is mainly used in the form of sheets or graphene oxide (GO). First records describing graphene came from 1917, when it was described in oxide form [9]. Not until 2004, did Novoselov and Geim manage to obtain graphene in its raw form of platelets, using mechanical exfoliation of graphite [10]. Since then, more methods of producing graphene have been designed, allowing for more efficient production with consistent properties of the final product. One of the novel methods is Flash Joule Heating (FJL) method developed by Luong et al. [11]. Graphene obtained through this method is called ‘flash graphene’ and is made by converting amorphous carbon using high voltage electric discharge to heat carbon powder up to 3000 K. The whole process takes place in a quartz tube, under atmospheric pressure or mild vacuum. Another method, with a possibility for industrial usage, is electrochemical exfoliation used by Krystek et al. [12]. Their method of electrochemical exfoliation of graphene allows for a higher production rate of ~200 mg of electrochemically exfoliated graphene per 180 min of the electrolysis process, compared to ~10 mg per 10 min for the classical electrochemical exfoliation process. Exfoliation of graphene, both mechanical with the usage of ultrasonication or electrochemical, is a process of peeling singular flakes out of a graphite block in a liquid solution. Addition of solvent prevents peeled flakes from accumulating.

**Figure 1:**
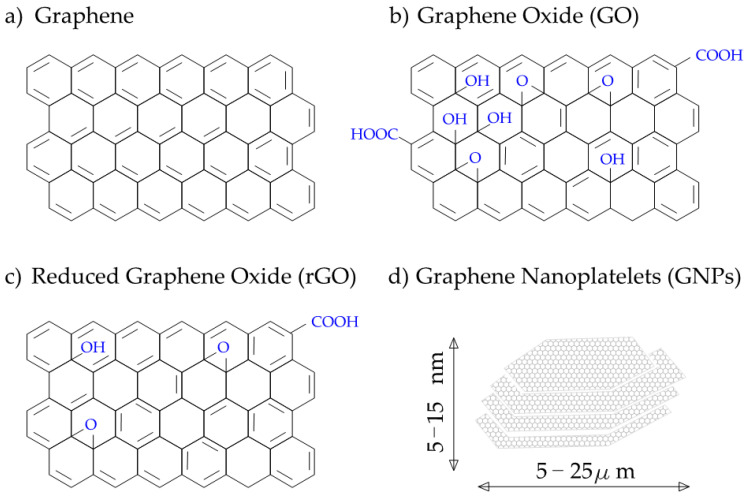
Representation of different forms of graphene materials: (**a**) pure graphene; (**b**) graphene oxide GO; (**c**) reduced graphene oxide rGO; (**d**) graphene nanoplatelets [14].

In its pure version, graphene appears as a 2D plane of carbon atoms arranged in a honeycomb shape. However, more functionalized versions have been developed. Graphene oxide (GO) is a more reactive derivative with multiple oxygen-containing functional groups. In terms of cementitious composites, those groups allow for easier dispersion in water however increase in conductivity of the composite is lower than for pure graphene [13]. Reduced graphene oxide (rGO) consists of fewer functional groups and is considered to be a middle ground between graphene and GO in terms of its properties [14]. The final group of graphene materials is graphene nano platelets (GNP), consisting of few stacked layers of graphene. The schematic representation of mentioned types of graphene is shown in Figure 1.

The excellent properties of graphene determine its position as one of the most promising and widely researched carbon nanomaterials. Its high thermal conductivity of ~5000 W/mK [15] and electron mobility of up to 200,000 cm^2^ V^−1^ s^−1^ [16] allow for its usage in self-sensing and energy-saving materials. At the same time, extraordinary mechanical properties: tensile strength of 130 GPa and Young modulus of 1 TPa [17], make graphene a viable material to be used as a reinforcing phase in composite materials.

### 2.2. Carbon Nanotubes (CNT)

**Figure 2:**
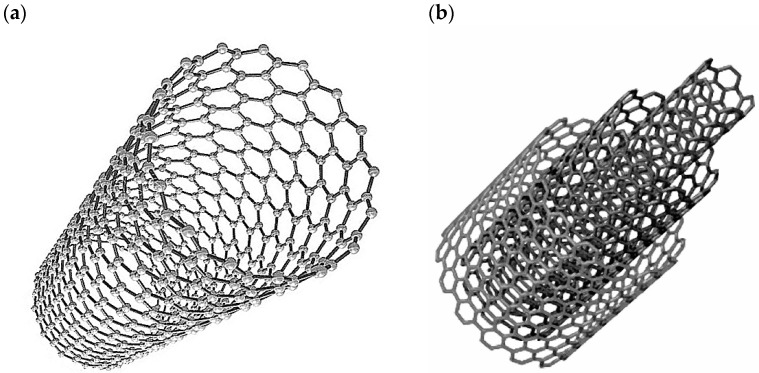
Schematic of carbon nanotubes structure: (**a**) single-walled nanotube; (**b**) multiwall nanotube [20].

Carbon nanotubes, first described by Sumio Iijima in 1991 [18], are classified as one-dimensional nanomaterial. Nanotubes consist of seamless, rolled graphene platelets, closed at the tip with half of the fullerene and are usually classified into one of the two groups: single-walled carbon nanotubes (SWCNT) or multi-walled carbon nanotubes (MWCNT) (Figure 2). The diameter of SWCNTs is between 1–2 nm, while for MWCNTs, which are stacked SWCNTs, 2–100 nm [19].

One of the main techniques used in the production of CNTs is chemical vapor deposition (CVD) which employs catalytical conversion of carbon materials. The carbon material is dissolved into individual atoms during the process and then rearranged to regrow in an elongated form. CNTs grow atom by atom into a tubular shape with the help of a metallic catalyst. CVD method is considered to be the most effective method of producing CNTs on an industrial scale and with sufficient stability of their properties [21].

Similar to graphene, CNTs possess excellent mechanical properties. Young modulus, measured directly by Wong et al. [22] reached values of 1.28 TPa, while most often it is assumed to be 1.0 TPa [23,24]. Tensile strength of SWCNTs is assumed to be in range up to 500 GPa [25], and for MWCNTs experimental results show values in the range of 11–63 GPa [26].

The electrical properties of CNTs are defined mainly by their elongated shape, which promotes the propagation of electrons along the tube’s axis. At the same time, their movement in other directions is restrained by layers of CNT itself. Therefore, CNTs exhibit high electrical conductivity of 10^6^ S/m for SWCNTs and >10^5^ S/m for MWCNTs [27] and have the highest current density of all known materials of 10^9^ A/cm^2^ [28].

The thermal conductivity of CNTs is tied to the phonon movement mechanism and highly depends on multiple factors, including its size and morphology [19,29]. Precise measurement of thermal conductivity in CNTs proves complicated and has been reported to be in the range of 2000–6000 W/mK [30,31,32].

### 2.3. Carbon Nanofibers (CNF)

**Figure 3:**
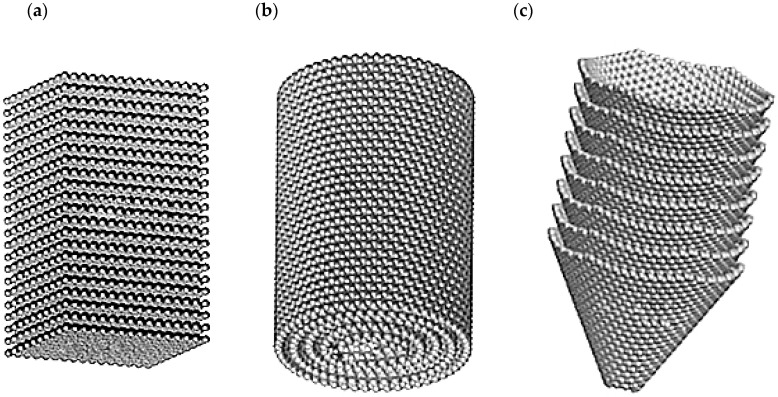
Typical shapes of carbon nano fibers: (**a**) platelet type; (**b**) tubular type; (**c**) fishbone type [33].

Carbon nanofibers are one-dimensional material consisting of stacked platelets or conical-shaped graphene. The overall shape of individual fibers depends on the type of catalyst used for growing the fibers. Typical shapes can be assigned to one of three groups (Figure 3): platelet, tubular, and fishbone [33,34]. The structure of CNFs allows for a wide range of modifications by combining them with other materials such as metallic nanoparticles, oxide alloys, or silica. This kind of modification allows for creating sensors capable of detecting various types of substances, including gases and organic matter such as bacteria or viruses. Typical dimensions of CNFs are 10 to 500 nm in diameter and ~10 µm in length [35].

The most common technique used for manufacturing CNFs is chemical vapor deposition coupled with thermal of plasma-assisted vapor deposition. Unlike CNTs, chemical vapor deposition of CNFs uses gaseous sources of carbon atoms. Decomposed by a high temperature, carbon atoms are grown into designated shapes by the use of a metallic catalyst [33,35].

Mechanical properties of carbon nanofibers, while presenting lower values than those of CNTs are still high with Young modulus in the range of 0.4–0.6 TPa and tensile strength of up to 7 GPa [25].

Similar to CNTs, CNFs possess excellent conductive properties due to their elongated shape, which promotes electron movement along the normal axis and the tunneling effect [34].

### 2.4. Summary of Carbon Nanomaterials

Presented carbon nanomaterials are most commonly used in nano-carbon cementitious composites. While all of them possess excellent mechanical, electrical, and thermal properties, each of them is excels in different applications. Individual features of each of the material provide them with certain advantages over the others. The flattened shape of graphene and its derivatives seem to perform best in promoting hydration, therefore vastly improving the microstructure and tightness of the composite. The elongated, closed shape of CNTs—along with their excellent conductivity—makes them the best choice in terms of conductive smart materials and strain sensors. Carbon nanofibers, while also having a beneficial, elongated shape, are more often used in biomedical applications because of their ability to be easily modified and functionalized to work with various types of biological substances. The detailed influence of carbon nanomaterials on cementitious composites will be discussed in Section 3.

Due to variations in methodologies and difficulties in direct measuring of properties of materials in nanoscale, some of the presented properties tend to have significant range of values. It is clear that more precise and unified methods need to be developed in order to assure better ways to compare the performance of individual types of materials and their final quality. The selection of test results for material properties is presented in Table 1.

## 3. Properties of Carbon Cementitious Composites

As discussed in the previous paragraph, carbon nanomaterials possess a number of unique properties, both mechanical and physical. Most of those properties can be exploited in order to enhance otherwise brittle and non-conductive cementitious materials to create stronger, functional composites. The following sections will provide insight into the most important properties of cementitious composites granted by the addition of carbon nanomaterials.

### 3.1. Hydration and Workability

Due to strong van der Waals forces between individual particles, carbon nanomaterials tend to accumulate into flocs. This phenomenon, along with a large surface area, causes water molecules to be trapped or absorbed by the nanomaterial, resulting in a reduction in workability. Workability is referred as an ease of flow and pouring of cementitious material. According to standards for cementitious mortars and concrete, workability is measured through comparing the diameter of pool created by a slump test. In this test, a specified amount of mortar of concrete is poured into a cone, then the cone is removed allowing for free flow of the material. Results by Jing et al., obtained from the mini-slump test, are showing that the addition of 0.4% of the binder weight (wt.%) of graphene can reduce workability by 39%. Additional SEM tests have been performed to exclude the influence of poor dispersion on the results [36]. Shuang et al. [37] observed a 40.6% increase in viscosity of cement paste with 0.09 wt.% of graphene by using the viscometer method. They have also ensured low influence of dispersion using UV–absorbance spectroscopy. A study by Konsta-Gdoutos et al. [38] showed an increment in viscosity for cement pastes with the addition of 0.08 wt.% of CNTs. They have also investigated the influence of surfactants and their concentration on the viscosity of the paste. It has been shown best results for a ratio of 5 surfactants to CNT, however, the most optimal ratio, regarding other properties, has been found at 4. Zou et al. [39] estimated the workability of cement paste with 0.038 wt.% and 0.075 wt.% of CNT using the mini-slump test. The decrease of workability was closely related to ultrasonication energy applied at the stage of CNT dispersion.

The workability problem can be mitigated by ensuring the proper dispersion of nanomaterials. If the material is more dispersed, the effects of water adsorption are distributed more evenly among the volume of material. The addition of superplasticizers and ultra-sonication can also efficiently reduce the effect of carbon nanomaterials on the workability of cementitious materials. It is assumed that it can be increased back to levels close to plain cementitious materials [37,38,39,40,41].

With large specific surface area and water absorption abilities, carbon nanomaterials might accelerate the hydration process through the nucleation effect. Baomin and Shuang [42] reported that the addition of graphene nanoplatelets by 0.06 wt.% exhibit a positive effect on the early stages of the hydration process. Such conclusions are supported by increased hydration heat recorded with isothermal calorimetry curves. Sun et al. [43] compared the degree of hydration of oil well cement doped with cellulose nanofibers and a mix of cellulose nanofibers and GNPs. They have found the best degree of hydration with a mix containing GNPs. The beneficial nucleation effect has also been reported with CNTs [44,45]. However, other studies suggest that higher amounts of CNTs might actually inhibit the hydration process by separating cement molecules [46]. Therefore, it can be concluded that a certain dosage of carbon nanomaterials promotes the hydration process, densifying material structure.

### 3.2. Microstructure

**Figure 4:**
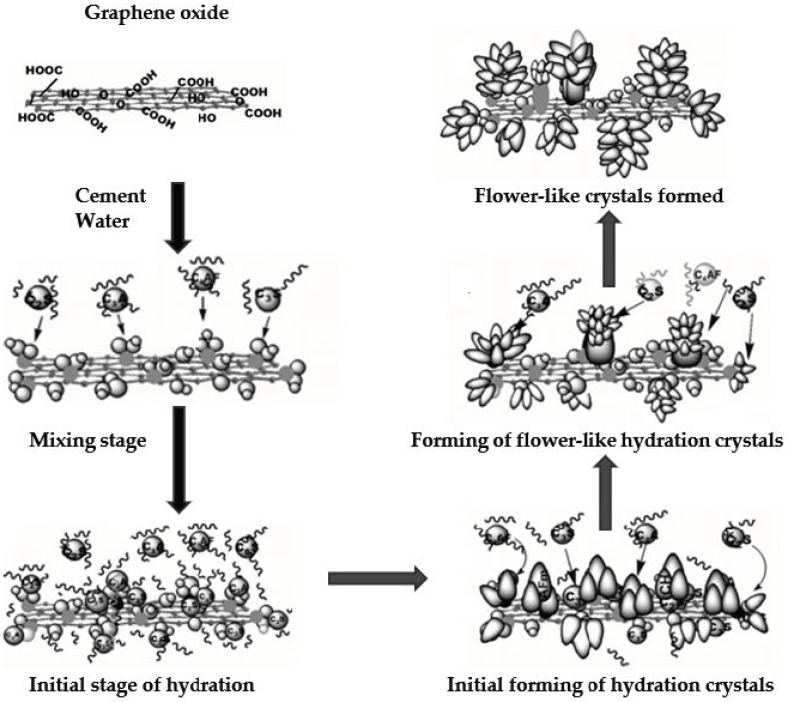
Schematic representation of the growth of hydration crystals on graphene oxide [47].

The small size of nanomaterials can contribute to improving the microstructure of cementitious materials. Nanomaterials can fill in gaps between C-S-H phase formations, and while enhancing the hydration process, they promote the growth of high stiffness C-S-H phase [12,22,38,47,48]. This is achieved through the nucleation effect, which causes cement molecules to be attracted to carbon nanomaterials. It is especially well observed with graphene oxide, thanks to the presence of multiple functional groups. As seen in Figure 4, they might promote growth of hydration products into crystal-like formations. [47].

An increase in hydration crystals and their concentration around nanomaterials leads to denser structure and lower pore size on a micro-scale. The results of structure densification can be observed via a scanning electron microscope (SEM) [47,48].

This kind of refined structure reduces penetration depth for water and aggressive substances, increasing the material’s durability and reducing permeability. Du et al. [49] investigated this mechanism through a rapid chloride penetration test. They have observed a reduction in water permeability, chloride diffusion and chloride migration coefficients by 80%, 80%, and 40%, respectively, for concrete with the addition of 1.5 wt.% of graphene and after 14, 56, and 90 days of immersion in a salt solution. In the cited research, chloride migration coefficient, is a coefficient derived directly from rapid chloride penetration test and is calculated using test parameters including voltage, test time, size of samples, and mean depth of chloride penetration assessed through splitting the sample and spraying it with AgNO_3_ solution. Chloride diffusion coefficient is a coefficient that is empirically derived, based on chloride diffusion curves. These curves are derived using measured concentration of chlorides on the surface of the sample and consecutive depths of the sample. The diffusion coefficient is then computed by fitting the curve to acquired measurement results.

### 3.3. Mechanical Properties

Carbon nanomaterials are considered reinforcing phases in cementitious composite materials. Their influence on material strength can be attributed to the aforementioned densifying of microstructure, strong bonding between nanomaterials and cementitious matrix, bridging of nano-sized cracks. Most of the test methodology for assessing cementitious composite’s mechanical properties is derived from both mortar and concrete standard tests as they are a viable way to compare results. For tensile strength, a direct uniaxial tension test on cylindrical or dog bone type of specimen is used. Most of the time, the mechanism of failure for composites is the same pure cement materials. For the same reasons, shapes and dimensions of samples match with corresponding samples advised in mortar and concrete standards respectively. To avoid deviations in results caused by cement additives, ordinary Portland cement is used in tests. Examples of selected experimental results for improvement of compressive, tensile, and flexural strength are summarized in Table 2.

The Young modulus of carbon nano-infused cementitious composites is improved through the increase in the amount of high stiffness C-S-H phase. Studies show an increase in the value of Young’s modulus by 35% [23] and up to 227% [57]. For CNF reinforced materials, Young modulus increase has been found to be around 50% [54], as for GNP, Horszczaruk et al. [56] reported an increase in Young’s Modulus value of around 100%.

### 3.4. Electrical Properties

The extraordinary electrical conductivity of carbon nanomaterials can contribute to increasing the conductivity of cementitious composites. Plain cement has a very low electrical conductivity and is considered close to insulators [58]. Including nanomaterials create conducting paths inside the structure of the material. Introduction of electrical conductivity in cementitious materials allows for creation of smart materials, mainly as strain sensors using piezoresistive effect and energy harvesting materials. These functionalities will be covered in more detail in Section 5 of the paper.

There are three main mechanisms for conduction in cementitious nanocomposites. Two of them are related to nanofiller itself. Contact conduction appears simply when individual conductive elements physically contact each other. The other mechanism is tunneling conduction which appears between materials that are close to each other but not in direct contact. It is induced by a strong electrical field appearing around strongly conductive materials, especially those with elongated, fine morphology, e.g., CNTs. The last of the conduction mechanisms is ionic conduction which also appears in plain cementitious materials as it is related to ions dissolved in pores of the material. Generally, this mechanism is relatively weak, especially in dry conditions, therefore, having a small contribution to the overall material’s conductivity [59,60].

A different approach has been taken by Soliman et al. [61] who attempted to describe conductivity in cementitious materials through mechanisms analogues with diffusivity, linking the conductive mechanisms with tortuosity of conductive paths inside the composite. They have combined their findings into a theoretical dissipation–tortuosity model and stated, that efficiency of smart composites can depend on conductor concentration and electrical tortuosity of the composite.

An increase in conductivity is closely connected with the amount of nanomaterial added and its dispersion. It has been proven that increasing the dosage of carbon nanomaterials effectively increases the material’s conductivity. The conductivity of nanocomposite is often described by percolation theory. The amount at which conductivity is no longer increasing is known as the percolation threshold. Reaching or exceeding this threshold means that the concentration and uniformity of distribution of conductive carbon nanoparticles are enough to create a continuous network of conductive paths [25,62].

The exact value of the percolation threshold is highly dependent on material type as well as the dispersion method. Du et al. [63] investigated the percolation threshold for GNP infused mortars and found it to be between 2.4 vol % and 3.6 vol %. D’Alessandro et al. [64] investigated a similar problem for MWCNT composites and found the percolation threshold to be 1 wt.% for either cement paste, mortar, and concrete.

Yoo et al. [65] investigated differences in electrical conductivity between CNTs, graphene, and graphite nanofibers at a constant value of 1 vol % for cement paste. The best results in terms of stable, undisturbed response have been achieved with MWCNTs.

The conclusion can be drawn that the previously mentioned elongated shape of CNTs works in their favor not only when considering the conductivity of plain nanomaterial but also in combination with cementitious materials. At the same time, graphene, while having excellent electrical properties in pure form, performs worse in creating conductive paths in cementitious matrix.

## 4. Dispersion

Dispersion of carbon nanoparticles and their influence on the properties of cementitious composites are important and widely discussed issues [23,39,40,41,45,66,67,68,69,70]. Due to strong van der Waal’s forces between individual particles and the hydrophobic nature of carbon nanomaterials, if left untreated, they have a tendency to agglomerate into larger flocs. Such agglomeration has a negative impact on all the abovementioned properties that cement nanocomposites might have. In terms of mechanical properties, large flocs of reinforcing material can create local defects in the overall structure of the composite and reduce the nano-reinforcement effect, as well as creating areas with non-uniform distribution of strength. Concerning conductivity, agglomerated nanomaterials will not allow for even distribution of conductivity along the volume of the material, therefore creating noises and errors in the recorded signal.

Meng et al. [53] tested various methods of dispersing CNFs in ultra-high performance concrete and its influence on mechanical properties. They have compared flexural strength of four samples: with no treatment; with mechanical stirring; with water reducer; acrylic acid and mechanical stirring and water reducer; acrylic acid and sonication. Results showed 65% improvement in flexural strength for the fourth specimen compared to one with no treatment, which demonstrates the importance of proper nanomaterial dispersion for achieving optimal strengthening.

Currently, there is no uniform method of assessing the proper dispersion of the nanomaterials. Most of the time, various spectroscopic methods are used to assess dispersion in aqueous suspensions or hardened composite itself. The scanning electron microscopy (SEM) method can also be employed; however, it allows for assessment of singular surfaces of the material.

The most popular dispersion techniques consist of adding chemical surfactants, modifying the surface of nanomaterials and usage of high-energy ultrasonic waves to split material particles forcefully.

### 4.1. Dispersion by Surfactants

Usage of surfactants employs chemical solutions to push nanoparticles away through electrostatic repulsion forces.

Vaisman et al. [71] pointed out not only the importance of proper dispersion of CNTs but also that noncovalent methods, with usage of surfactants, from among all other, provide a stable and safe way of dispersion with no risk of damaging the CNTs.

Rastogi et al. [72] attempted to compare the efficiency of different surfactants on dispersion of CNTs in water. They have compared four surfactants: Tween 20, Tween 80, Triton X100, and sodium dodecyl sulphate (SDS). For each of the chemicals the optimal dosage has been established. The dispersion was assessed by independent testing with UV–vis spectroscopy and transmission electron microscopy. The best results have been achieved with SDS and Triton X100 surfactants while the main conclusion of the research was the importance of exact ratio of CNTs and surfactant. It was observed, that using lower of higher than optimal dosages of surfactants showed noticeable deterioration in dispersion quality. Research by Wen et al. [73] show similar results in effectiveness of SDS surfactant. Besides laboratory testing they have estimated binding energy between SDS molecules and CNTs to calculate the most optimal dosage of the surfactant. A similar approach by Poorsargol et al. [74] was used to compare effectiveness of SDS and cetyltrimethylammonium bromide (CTAB) surfactants on graphene flakes dispersion. They have also combined experimental and molecular dynamics approach to model the behavior of surfactant molecules and graphene. When used solely, SDS proved better than CTAB, however the best results overall have been achieved with mix of both substances.

For dispersion of graphene, Smith et al. [75] compared the performance of several ionic and non-ionic surfactants, most of them already known for good results in dispersing CNTs. The dispersion was assessed with UV–vis spectroscopy and zeta potential measurements right after adding the surfactant and after a week time. The final results, concerning also final flake size and thickness showed best results for SDS and sodium cholate (SC). Moreover, it has been stated that for ionic surfactants the concentration is dependent on zeta potential of surfactant-coated graphene sheets while for non-ionic surfactants concentration scales with repulsive potential barrier.

Superplasticizers, commonly used as water-reducing reagents in concrete, have been reported to perform well in the role of surfactants. Metaxa [66] reported efficient dispersion of exfoliated graphene by the usage of poly-carboxylate superplasticizer. Du and Pang [40] found an optimal superplasticiser dosage to be 15% of the mass of GNP. Kim et al. [67,70] added silica fume along with superplasticizer to improve dispersion of CNTs. They observed that fine, round particles of silica fume could mechanically separate CNTs, therefore improving their dispersion with no significant effect on the composite’s conductivity and microstructure.

Moreover, silica fume further reduced the porosity of cementitious composite. D’Alessandro et al. [69] investigated a new type of surfactant, called DDA, developed at their lab and dedicated to the dispersion of CNTs. They have proven that DDA dispersant is a stable solution and improves the conductive properties of CNT composites compared to commercially available products.

### 4.2. Sonication

Ultrasonication is often used along with the addition of surfactants to achieve optimal results in terms of nanomaterial dispersion. It employs high-energy ultrasound waves with a frequency over 20 kHz to agitate the particles and separate them by peeling them off one another [76]. Du et al. [40] found the optimal amount of sonic treatment to be 1 h per 6 g of GNP and with a power of 210 W. Such treatment retained GNPs in suspension for 6 h. Zou et al. [39] compared CNT dispersion treated with different levels of ultrasonication energy. They found that higher energy is needed for a higher dosage of CNTs. The energy required to disperse CNTs uniformly was measured as 250 J/mL for 0.188 wt.% of CNTs.

## 5. Applications of Cementitious Nanocomposites

Research in the field of functional composite materials revolves mainly around creating smart and functional materials. Those materials are engineered to provide additional functionality in terms of monitoring of its strains, energy harvesting, electromagnetic shielding, or self-healing. They can be implemented as a part of structural members, insulation, strengthening, or façade elements.

Combined with solutions in the area of data processing novel materials can be incorporated into concepts of smart structures or smart cities. Data gathered from smart sensors can be stored in central databases and computed using artificial neural networks (ANN) to provide a real-time status assessment of infrastructure [77]. The web of mutually connected sensors can act as a neural system of monitored structure, reaching its every part while remaining closely connected to the base material forming the structure.

### 5.1. Piezoresistive Sensors

Cementitious strain sensors, using piezoresistive principle, attract major attention among researchers [51,52,63,64,69,78,79,80,81,82,83,84,85,86,87,88,89,90,91,92]. The main research scope of this kind of sensors is strain sensing for maintaining existing structures, especially critical infrastructure or historical objects. Weight in motion sensors [79] and dynamic sensors [80,81,83,93] are also subject of research in the area of smart sensors.

Piezoresistive sensors base on the principle that ties fractional change in resistance of the material with the state of its strain. In cementitious nanocomposites, it is achieved through principles tied to their conductivity. Under strain, the distance between conductive filler particles changes. This affects both contact and tunneling conduction mechanisms, therefore changing the resistivity of the material. Under tensile strains conductivity decreases because particles that were in direct contact are separated, or the distance between them increases, disabling the tunneling effect. Under compressive strains, particles are moving closer to each other, new spots of direct contact appear, and more of the particles are in the tunneling effect range; therefore, overall conductivity increases. The sensitivity of this mechanism is highly dependent on the amount of nanomaterial included in the composite. Lower dosages might exhibit higher sensitivity, especially under tension, because conductive paths between carbon nanoparticles will be cut off sooner. Too high a dosage of nanomaterial can render sensors ineffective since strain level causing noticeable changes in their resistivity might be too high, close to the ultimate strain of the cementitious material.

Sun et al. [51] tested the behavior of composites with 5 vol % multi-layer graphene under cyclical load reaching up to 20 MPa. Their results show consistent and clear changes in fractional resistivity along with stress, even after multiple cycles with changing frequency. Frąc and Piechór [78] measured fractional change in resistivity (FCR) for different dosages of expanded graphite. They have received the best results of 9.8% maximum change in resistivity for composites with 5 wt.% inclusion of expanded graphene while maintaining a clean plot of FCR against stress.

Dong et al. [92] conducted research on the piezoresistive behavior of composites containing GNPs and silicone hydrophobic powder (SHP). The main scope was to assess the effect of water absorption on the piezoresistivity of the material. For unsaturated samples with 1 wt.% and 2 wt.% of GNP and the same amounts of SHP, the fractional change of resistance showed linearity in change under the rising magnitude of the load. Under water immersion, readings have been disturbed for samples not containing hydrophobic layers of SHP and were stable for samples with SHP.

Galao et al. [91] fabricated strain sensors with CNFs attached to the concrete specimen’s surface. With a generally good trend of sensing abilities, problems with strain transfer occurred. Moreover, CNF sensors became less accurate in the low strain range and sensitivity increased as strains progressed.

**Figure 5:**
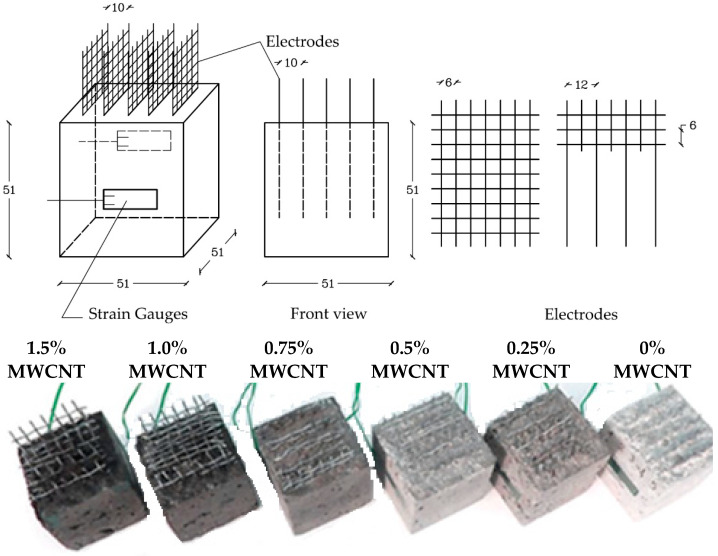
Example of embedded CNT strain sensor [80].

A similar approach has been taken by researchers from The University of Perugia [65,70,81,84,85], who researched applications of premade sensors embedded into concrete elements (Figure 5). Their work revolved mainly around CNT composites and scoped on studying the influence of dispersion and contents of CNTs on piezoresistive behavior of the composites, going as far as creating a dedicated surfactant that proved to further increase the conductivity of the material [69]. Thanks to those calibrations, embedded sensors prove the ability to detect both static and dynamic stresses with high accuracy [83,84]. Another approach to sensing dynamic effects has been proposed by Rao et al. [93]. Their proposition of piezoresistive sensor included the usage of pure and functionalized CNTs. Tests have been performed on a medium-scale model of a concrete bridge beam, in which vibrations were induced via a pulsing hammer.

Additionally, a cyclic compression test has been performed to assess the strain sensitivity of the sensors. Compared to standard accelerometers, CNT sensors showed highly accurate results for the first three modes of vibration. The best results have been obtained for the sensor with 0.75 wt.% of CNTs functionalized with the carboxyl group.

An interesting approach was taken by Liu et al. [94], who created CNTs coated with nickel layers. This treatment allowed for orienting CNTs inside of composite using a magnetic field. Tests for piezoresistive behavior showed that composites containing 1.2 vol % of Ni-CNTs oriented to be parallel to the magnetic field show a high gauge factor of 993.

Jung et al. [82] investigated possibilities of field casting ultra-high performance concrete (UHPC) with the addition of CNTs and steel fibers. Results of electrical tests showed a 10-times higher gauge factor of UHPC with steel fibers and CNTs than UHPC containing just steel fibers. Additionally, CNTs reduced the voltage needed for the electrical curing of concrete, making it a viable solution for field casting.

Attempts have been made to create cementitious sensors with the hybrid composition of carbon materials. Azhari and Banthia [87] tested cement sensors with 15 vol % of carbon fibers and 1 vol % of CNTs. Results obtained by them show an apparent decrease in resistivity that occurs with micro-cracks under compressive strains. Research by Lee et al. [88] give similar results in terms of the optimal ratio of CNTs however, their focus was on optimizing carbon fiber and CNTs proportions to create more economical sensors with comparable performance to clear CNT sensors. Their results show that a sensor with 0.1 vol % of carbon fibers and 0.5 vol % of CNTs has similar sensing capabilities as a sensor with 1% CNTs while costing half its price. Another approach to the hybrid material sensor was made by Abedi et al. [90], who combined CNTs and GNPs. According to their study, the sensor with 0.5 vol % of both CNTs and GNPs can achieve a gauge factor of 460 under compression, which is nearly twice as much as results gained from individual nanomaterials.

One of the most common ways of expressing the effectiveness of piezoresisitve sensors is fractional change in resistivity (FCR) which is a percentage ratio of change in resistivity over base resistivity of the material. Comparison of selected results of FCR are shown in Table 3.

### 5.2. Thermal Material

Carbon nanocomposites’ superior thermal properties can be exploited to create heating materials, energy harvesting, or heat sink.

Applying the Joule-heating mechanism allows for using carbon-infused materials for heating. According to the aforementioned law, the flowing current can generate heat and is proportional to the square of applied voltage. Kim et al. [95,96] experimented with heating materials containing CNTs. They have discovered that 0.6 wt.% of CNTs allowed for the stable generation of heat of up to 70 °C [95]. Further research consisting of both CNTs and carbon fibers has shown improved results while creating stable heat conducting paths along with the material [96]. Frąc et al. [97] used expanded graphite combined with paraffin and obtained promising results with 20 wt.% of graphite/paraffin cementitious composite, achieving heat generation of 25.7 kW/m^2^ with an applied voltage of 10 V. Moreover, the composite material was able to preserve heat for nearly 5 h after power was turned off.

Heating composites can also be applied as de-icing material for bridges and roads. Wu et al. [98] researched three-phase composite materials dedicated to de-icing. With the hybrid composition of 1 vol % steel fibers, 0.4 vol % carbon fibers and 4 vol % graphite, they have achieved an 8.7 °C increase in temperature after 2.5 h under 27 V and 21.8 °C after 2 h under 44 V. With stable results and high enough current density, the three-phase composite has been assessed as a satisfactory solution for pavement de-icing.

Energy harvesting is a concept of using small, natural portions of energy that are absorbed by building surfaces, especially in cities. Thermal energy, produced by sunlight and the urban environment, accumulates in structures and contributes to the Urban Heat Island effect [99]. Cementitious composites with graphene or graphite can be used to directly harvest extensive thermal energy in cities via the thermoelectric effect, therefore, reducing the overall temperature inside the buildings while harvesting energy in an environmentally friendly way. Ghosh et al. [100] proposed the usage of 15 wt.% graphene to achieve stable thermoelectric properties and a Seeback coefficient of 34 µVK^−1^ at 70 °C. Wei et al. [99] used expanded graphite with 15 wt.% to a similar effect.

### 5.3. Electromagnetic Shielding

Electromagnetic shielding is defined as reducing or attenuating a magnetic field at a point in space by inserting a shielding material between the electromagnetic field source and that point. It is measured with shielding efficiency (SE) in decibels and is a sum of initial reflection losses from the surface of the material and absorption and penetration loss within the material [101]. Historically metallic materials were used as EM shielding material due to their reflective properties. However, due to their susceptibility to corrosion and unwanted reflection phenomena, carbon materials have been considered as an alternative [101]. Known research in this area includes the usage of CNTs and graphene.

Nam et al. [102] tested composites with 0.6 wt.% of MWCNTs, fly ash and silica fume and achieved SE of 8–57.1 dB for frequencies in the range of 1–18 GHz. In terms of graphene materials, Cui et al. [103] achieved −5 dB reflection of EM waves for composites with 5 wt.% of NGPs. Sun et al. [51] tested composites with different volumes of multi-layered graphene and obtained best results of −7.7 dB to −33 dB of reflectivity for frequencies in the range of 4–8 GHz and 10 vol % of MLG.

### 5.4. Self-Healing

Self-healing is the ability to repair and close micro-cracks without human intervention attributed to concrete and other cementitious materials. Two main types of self-healing can be distinguished [104]. Autogenous healing consists of further hydration taking place in the concrete volume. This type of healing, even if using additions or nanofillers, relies only on unhydrated cement. Autonomous healing employs the usage of engineered solutions such as shape memory alloys, capsules, bacteria.

With regards to carbon nanomaterials, self-healing in cementitious material can be attributed to autogenous healing. Just as in the early stages of material hydration, carbon nanomaterials can act as nucleation sites promoting the regrowth of hydration products from unhydrated cement left in the volume. Siad et al. [105] experimented on concrete specimens with the addition of carbon fibers and CNTs. Pre-cracked samples were cured in water for 30, 60, and 90 days and recovery of their flexural strength was assessed. Final results showed that samples with 0.5 wt.% of CNTs recovered up to 5% of flexural strength, which proved self-healing ability to some extent. Öztürk et al. [106] tested the self-healing abilities of concrete specimens with PVA fibers, nanosilica and 0.55 wt.% of CNTs. Healing efficiency has been evaluated based on the comparison of electrical resistance of pre-cracked and healthy specimens as well as visual and SEM inspection. Results showed visually noticeable closure of cracks after 14 days for composites with CNTs, moreover, the amount of CaCO_3_ recreated during the healing period has been assessed as 27%.

## 6. Modeling of Carbon Cementitious Composites

With the rising popularity of carbon cementitious composites in research, a natural need for theoretical models appeared. As a relatively new kind of materials involving numerous phenomena on nano-level and multiple unknown regarding material orientation or dispersion, creating a reliable model of nanocomposites is difficult. Various models proposed in literature usually focus on just one aspect of the composites.

Park et al. [107] focused on modeling electrical properties of CNT composites. They have proposed a micromechanics model coupled with a particle swarm optimization algorithm to estimate the electrical conductivity of the composite. Calibrations of the model included conductivity measurement of cement elements with different parameters in terms of CNT and carbon fiber addition and water to cement ratio. Composite’s parts have been separated with regards to their material and assigned into phases. Then, phases have been homogenized using effective medium theory for cement, CNTs, and carbon fibers phases and Mori-Tanaka model for pores. The particle swarm optimization has been combined with micromechanics models to address different w/b, CNT, and CF content variables. Results acquired from the models have been compared with electrical measurements and SEM imaging to calibrate the model. The most decisive parameter for assessing the conductivity of the composite has proven to be the waviness of CNTs. This parameter has been evaluated based on SEM images of individual CNTs.

Eftekhari et al. [108] attempted to model mechanical properties and crack propagation in a concrete specimen with the inclusion of CNTs. They have adapted a multiscale approach with nanoscale for just CNTs, micro-scale for cement composite and mesoscale to assess concrete cracking using extended finite element method (XFEM) method. Each of the models was scaled up to include its influence on the larger scope of the whole model. Mechanical properties of CNTs have been simulated by the molecular dynamics method. Then, after upscaling those properties, hydration model has been used to assess the chemical composition of the composite. FEM with the assumption of an isotropic damage model was then used to acquire the mechanical properties of the cement-CNT composite. Those results were used for the XFEM model for general crack propagation computations.

Another example of multiscale approach for modeling cementitious composites has been employed by Wang et al. [109]. They have attempted to model hydration process of cementitious material with and without addition of CNTs. In microscale, they have modeled formation of hydration products considering reaction rate and kinetics laws which apply to small scale of hydration reactions. In mesoscale, mechanical properties of cement material have been evaluated in a volumetric way, taking into consideration volumes of hydration products, pores, and initial volume of water. Finally, in macroscale, mechanical properties of whole cementitious material have been evaluated, using micromechanics models and linked with degree of hydration and time. Results from each step have been up scaled and used in further considerations. Inclusion of CNTs has been modeled as random using probability density functions. In the final part, effect of CNTs on overall mechanical properties of the composite has been estimated. For a constant content of 5 vol % the effect of distribution, sample size, influence domain, and cementitious matrix itself have been evaluated. Final results have shown a good correspondence with experimental method.

Garcia-Macias et al. [110] considered a micromechanics model coupled with FEM analyses for modeling the response of CNT infused composite under 3D stresses. They have focused on differences in longitudinal and transverse directions of conduction for CNTs and parametric descriptions of conductive mechanisms concerning both contact and tunneling conductivity. In another work [111], they have also considered the orientation and shape of CNTs as a factor in the resistance model.

Kostrzanowska-Siedlarz [112] proposed a statistical model for predicting rheological parameters of mortars containing CNTs. Those parameters have been assumed according to Bingham rheological model, with all required values obtained from laboratory tests using a rheometer. Obtained results have been compared using variance analyses ANOVA in order to compare variances between different factors. In the next stage, Wilcoxon pair analyses were performed, and multiple regression models have been proposed.

## 7. Conclusions

Carbon nanomaterial cementitious composites remain an important focus of researchers around the world. Current advancements made in this field give the answer to key issues considering usage and a better understanding of nanocomposites. Despite numerous studies conducted on most types of nanomaterials, uniform rules, and models are still missing. With multiple factors contributing to each and every property of cement nanocomposites finding uniform solutions for dispersion, volumes, and composition of the composites is needed, to investigate mechanisms behind strengthening, piezoresistive, and thermal phenomena.

The main focus on current research is still revolving around mechanical, thermal and electrical properties of cementitious composites. These fundamentals require in-depth investigation in order to understand their mechanisms and be able to progress with practical applications of cementitious composites. The significant differences in terms of mechanical properties between researchers must be addressed and unified in order to precisely describe the causes of such differences. More precise ways of collecting and analyzing data from piezoresistive sensors are needed to provide more practical and reliable solutions. Moreover, increasing the efficiency and reduction of costs for both smart sensors and energy harvesting materials are needed in order to allow for large-scale usage of those materials. More practical and large-scale tests or real structures could also provide important insight in terms of applicability and behavior of functional materials in a real, uncontrolled situation.

Creating uniform and stable solutions for nanocomposites is needed to implement their practical applications on a broader scale. Structural health monitoring, energy harvesting, heating, and EM shielding systems need to be simplified and standardized in order to allow for industrial usage and wider impact on civil engineering solutions.

Table 4 summarizes, in authors opinions, the most relevant recent research on cementitious composites with carbon nanomaterials that were presented in previous sections of the paper.

## Figures and Tables

**Table 1:** Comparison of selected results for mechanical properties of carbon nanomaterials.

Property	Graphene	SWCNT	MWCNT	CNF
Tensile strength	130 GPa [17]	500 GPa [25]	11–63 GPa [26]	7 GPa [25]
Young modulus	1 TPa [17]	1.0 TPa [24]	1.28 TPa [22]	0.4–0.6 TPa [25]

**Table 2:** Selected results of mechanical properties improvement of cementitious composites for different types of nanomaterials.

Mechanical Property	Type of Composite	w/b	Increase	Material/Concentration	Reference
Compressive strength	Cement paste	0.45	28%	GO: 0.04 wt.%	Qureshi et al. [50]
	Cement paste	0.3	54%	Graphene: 2 vol %	Sun et al. [51]
	Cement paste	0.26	38%	GO: 0.05 wt.%	Madbouly et al. [52]
Tensile strength	Mortar	0.5	79%	Graphene 0.05 wt.%	Krystek et al. [12]
	UHPC	0.2	55%	CNF: 0.3 wt.%	Meng et al. [53]
Flexural strength	Cement paste	0.45	83%	GO: 0.04 wt.%	Qureshi et al. [50]
	Cement paste	0.5	45%	CNF: 0.048 wt.%	Konsta et al. [54]
	Cement paste	0.3	40%	MWCNT: 0.048 wt.%	Konsta et al. [23]
	Cement paste	0.4	269%	MWCNT: 0.2 wt.%	Al-Rub et al. [55]
Young modulus	Cement paste	0.3	35%	MWCNT: 0.048 wt.%	Konsta et al. [23]
	Cement paste	0.5	50%	CNF: 0.048 wt.%	Konsta et al. [54]
	Mortar	0.57	100%	GO: 3 wt.%	Horszczaruk et al. [56]

**Table 3:** Selected results of research on piezoresistive sensors. FCR—fractional change in resistivity.

Type of Composite	FCR	Material/Concentration	Reference
Cement paste	15%	Graphene: 5 vol %	Sun et al. [51]
Cement mortar	9.8%	Expanded graphite: 5 wt.%.	Frąc et al. [78]
Cement paste	~15%	GNP: 1 wt.%	Dong et al. [92]
Cement paste	6%	COOH-MWCNT: 0.75 wt.%	Rao et al. [93]
Cement paste	23.75%	Ni-CNT: 1.2 vol %	Liu et al. [94]
UHPC	25%	CNT: 1.2 wt.%	Jung et al. [82]
Cement mortar	~15%(compression) ~3%(flexural)	CNT: 1 wt.% + GNP: 1 wt.%	Abedi et al. [90]

**Table 4:** Summary of the most relevant research on functional cementitious composites presented in the paper.

Area of Research	Subject of Research	Authors
Piezoresistive sensors	Self-sensing material	Sun et al. [51]
		Frąc et al. [78]
		Dong et al. [92]
		Abedi et al. [90]
		Liu et al. [94]
		Jung et al. [82]
	Embedded sensor	D’Alessandro et al. [84]
		Rao et al. [93]
	Embedded sensor + dispersion	D’Alessandro et al. [69]
Thermal material	Heating/heat storage material	Kim et al. [96]
		Frąc et al. [97]
	Energy harvesting	Ghosh et al. [100]
		Wei et al. [99]
Electromagnetic shielding	Shielding material	Nam et al. [102]
		Cui et al. [103]
		Sun et al. [51]
Self-healing	Autogenous healing material	Siad et al. [105]
		Öztürk et al. [106]
Modeling	Material morphology	Park et al. [107]
	Mechanical properties	Eftekhari et al. [108]
	Conductivity model	Garcia-Macias et al. [110]
		Garcia-Macias et al. [111]
	Statistical model for rheological parameters	Kostrzanowska-Siedlarz [112]

## Data Availability

No new data were created or analyzed in this study. Data sharing is not applicable to this article.
